# Highly sensitive and selective colorimetric sensing of CO_2_ for biomedical applications

**DOI:** 10.1007/s13205-022-03396-9

**Published:** 2022-10-31

**Authors:** Shahina Shahid, Mithra Geetha, Kishor Kumar Sadasivuni, Divya Remani, Suresh Muthusamy, Asan G. A. Muthalif, Somaya Al-maadeed

**Affiliations:** 1 Center for Advanced Materials, Qatar University, P.O. Box 2713, Doha, Qatar; 2 Department of Electronics and Communication Engineering, Kongu Engineering College, Erode, Tamil Nadu India; 3 Department of Mechanical and Industrial Engineering, Qatar University, P.O. Box 2713, Doha, Qatar; 4 Department of Computer Science and Engineering, Qatar University, P.O. Box 2713, Doha, Qatar

**Keywords:** Carbon dioxide, Colorimetry, Biomedical applications, Multi-dye, Biomarker

## Abstract

The concentration of carbon dioxide (CO_2_) in unhealthy people differs greatly from healthy people. High-precision CO_2_ detection with a quick response time is essential for many biomedical applications. A major focus of this research is on the detection of CO_2_, one of the most important health biomarkers. We investigated a low-cost, flexible, and reliable strategy by using dyes for colorimetric CO_2_ sensing in this study. The impacts of temperature, pH, reaction time, reusability, concentration, and dye selectivity were studied thoroughly. This study described real-time CO_2_ analysis. Using this multi-dye method, we got an average detection limit of 1.98 ppm for CO_2_, in the range of 50–120 ppm. A portable colorimetric instrument with a smartphone-assisted unit was constructed to determine the relative red/green/blue values for real-time and practical applications within 15 s of interaction and the readings are very similar to those of an optical fiber probe. Environmental and biological chemistry applications are likely to benefit greatly from this unique approach.

## Introduction

Human exhaled breath can be a useful indicator of a respiratory system problem. It contains inorganic molecules like oxygen, nitric oxide, carbon dioxide, and non-volatile substances like cytokines, leukotrienes, isoprostanes, and H_2_O_2_ as volatile organic compounds (VOC). Saturated hydrocarbons may be included in certain VOC. Sulfur, nitrogen, and oxygen are included as unsaturated hydrocarbons. Most breaths contain acetone, isoprene, methanol, ethanol, and other alkanes and alcohols (Lin et al. 2020). An average adult's exhaled breath contains 35,000 to 45,000 parts per million (ppm) of CO_2_, 100 times greater than what is found in the outside air (OSA) (Geetha et al. 2022). Abnormal CO_2_ levels could indicate an electrolyte imbalance in the body or difficulty in breathing. CO_2_ levels in the body that are too high or too low can suggest a range of health problems. In the intensive care unit, surgical room, critical care unit, neonate intensive care unit, and clinical environment, human CO_2_ helps doctors identify extubation outcomes, spot ventilation derangements, bronchospasm, and therapy effectiveness (Rasera et al. 2015; Jaffe and Orr 2010). To check and diagnose cardiopulmonary diseases like asthma, pulmonary embolism, COPD, pneumonia, and congestive heart failure, CO_2_ features like EtCO_2_, time spent at EtCO_2_, exhalation duration, respiratory rate, Hjorth parameters, area ratio, slope ratio, and end-exhalation slope can be used (Brown et al. 2013; Fabius et al. 2016; Maestri et al. 2013).

Many health issues are related to carbon dioxide (CO_2_), and hence CO_2_ detection with high accuracy and quickness is very important (Zhao et al. 2014). For example, end-tidal CO_2_ (EtCO_2_), which measures CO_2_ concentration at the breath ends, provides a non-invasive evaluation of the physical condition and hence a non-invasive method to diagnose chronic obstructive pulmonary disease (COPD), asthma, and cardiovascular disease (Mannino et al. 2002). Yaron et al. (1996) studied the alveolar slope parameter and measured the slope for 5 expired breaths in 18 asthmatic participants in a study for asthmatic patients before and after medication. Since then, it's been clear that the capnogram can be used to track inflammatory states in cardiopulmonary illnesses. Guthrie et al. (2007) used a microcap plus device (Oridion Capnograph, Israel) to measure EtCO_2_ in 16 asthmatic patients and concluded that non-invasive EtCO_2_ assessment at the bedside is possible in children with acute asthma. Nagurka et al. (2014) researched the effects of extreme (low and high) EtCO_2_ on 299 asthmatic patients. They suggested that early EtCO_2_ measurements might be used as a biomarker for asthmatic symptoms outside of the hospital setting. Furthermore, Kesten et al. (1990) looked at the RR association between 42 non-asthmatic patients and 47 asthmatic patients who were acutely unwell and concluded that during asthma attacks RR rises, consistent with Tidemandsen et al. (2021) and Kassabian et al. (1982).

CO_2_ in breath is currently measured using infrared detection technologies. While useful, humidity, present in both breath and air, causes significant interference with this technology. Furthermore, the infrared technique necessitates particular sample pre-treatments to minimize humidity, which increases the device's cost and limits its applicability in clinical settings (Zhao et al. 2014). As a result, a low-cost, simple-to-use, compact and accurate CO_2_ sensor is needed to track CO_2_ in human breath (Kumar and Krishnamoorthi 2021; Danckwerts et al. 1963). There are numerous CO_2_ analytical techniques in use today (Humphreys et al. 2021; Ottonello-Briano et al. 2020; Kostenko et al. 2021). To measure CO_2_, the capnograph instrument is widely used nowadays. A capnograph, or continuous plot of exhaled CO_2_ over time, uses infrared technology for non-invasive monitoring of CO_2_ in human respiration. However, because capnography is performed in an offline manner, these capabilities have not been included in capnography in a clinical environment. Furthermore, it is costly and inconvenient and has a poor estimation of lung ventilation and perfusion state (Williams et al. 2021). As a result, a lightweight, low-cost, precise, and quantitative CO_2_ measurement instrument is urgently needed. So, we present real-time CO_2_ monitoring equipment for human respiration that can be utilized for cardiorespiratory assessment in a user-friendly setting.

A detection method based on colorimetry, which recognizes CO_2_ based on the change in color of a pH-sensitive indicator, is an alternative to infrared sensing (Nakamura and Amao 2003; Elosua et al. 2006; Fernandez-Sanchez et al. 2007; Shivananju et al. 2013; Carvajal et al. 2010; Qamar et al. 2021). The colorimetric technique offers several advantages over infrared CO_2_ sensors, including simplicity, miniaturization, low cost, and humidity resistance, making colorimetric sensors an appealing technology. These sensors are appealing because they are often inexpensive, and fiber optics can be utilized as remote sensors. This opens the way for developing a new sensor range that can perform various in vivo and in vitro clinical evaluations (Humphreys et al. 2021). We examined a reversible colorimetric sensor based on pH and CO_2_ determination. The current device employs multi dyes to precisely monitor CO_2_ levels in humans at varying temperatures. Other than biomedical uses, CO_2_ detectors can be used for indoor air quality monitoring, landfill control, process control, and controlled environment horticulture.

## Materials and methods

### Materials required

CO_2_ gas, benzene (37% w/v), ammonia (25%), formaldehyde (99%w/v), ethanol (99.5%), hydrogen peroxide (30%), neutral red, and phenol red (2%), and m-cresol purple were obtained from Sigma-Aldrich, toluene (36–40% w/v), sodium hydroxide, and hydrochloric acid. All the chemicals used in the experiment were of analytical grade. Purified water for the experiment was supplied by the Millipore Milli-Q water system. A Biochrom UV–vis spectrophotometer carried out UV–vis spectroscopy characterization with a scanning range of 190–1100 nm. The dye samples were scanned at a medium scan speed in the 300–700 nm range. For characterization, a bandwidth of 2 nm and a step input of 1 nm was used.

### Methods

#### Dye preparation and characterization

The studies were carried out using the solutions of 0.02 mM Neutral red, 0.01 mM Phenol red, and 0.07 mM m-Cresol purple. A UV spectrophotometer was used to perform the characterizations at ambient temperature. A graph between absorbance and wavelength was created after characterization and data assessment.

#### Optimization of kinetic parameters

The dye solutions were adjusted to acidic (4), neutral (7), and basic (10) pH to analyze how pH affected the results after the addition of 50 ppm CO_2_. After passing CO_2_, the solutions were heated to 50 °C to see if the dye system could be reused. Different concentrations of CO_2_ were added to the dye solution at room temperature and a certain pH to optimize the reaction condition. The dye solution was heated at different temperatures, and the temperature effect was also studied at specified pH. For selectivity analysis, 1 mL of 5 ppm ammonia, benzene, ethanol, ammonia, hydrogen peroxide, toluene, and formaldehyde was added to the dye solutions at room temperature and was observed for color change. The difference in absorbance was noted for understanding the concentration of the test solution, the temperature of the dye–biomarker mixture, pH, and the selectivity of the dye solutions.

#### Development of smartphone-assisted sensor prototype

IoT-based colorimetric sensor prototypes can be easily fabricated at a low cost. Using open-source hardware and software solutions, we developed a 3D printable, open-source colorimeter. Colorimeter detector assemblies are divided into the following three independent regions, namely the light source area, sample area, and color detector area. LEDs were used as the light source, which consisted of the following four different colors: red, blue, white, and green. After the light source area, there were three chambers dedicated to the sample zone. Each section was divided into three sections containing three cuvettes containing individual dye solutions. A 3D-printed protective case protected the sample zone, which was immediately followed by a detector zone with a color detector. To maximize repeatability, the light source, sample zone, and sensor were horizontally aligned, and the prototype was painted black to minimize reflections. Three cuvettes containing dye solution were placed in series one after another and were illuminated by the LED source. The color detector then intercepted the light transmitted from the sample zone. By analyzing the transmitted light, the detector represents the RGB values. When exposed to biomarkers, the dye solution changes color. With the sensor prototype connected to a device (LCD/smartphone) via Bluetooth, the detector identified color changes in dye solutions and displayed unique RGB values.

## Results and discussion

CO_2_ (concentration ranging from 50 to 120 ppm) was added to dye solutions in an acidic, basic, and neutral medium and examined for any visible color change to create a highly sensitive colorimetric CO_2_ sensor. Furthermore, the dyes' response time, pH impact, temperature effect, concentration effect, and selective nature were investigated and assessed. Table 1 shows the list of notations that were utilized. P stands for pH, whereas x stands for a specific pH, T stands for temperature, and y stands for a specific temperature. The concentration of the analyte is denoted by the letter Z.

**Table 1:** List of notations used

Dye	Indication of dye	Specific pH (Px), temperature (Ty) in °C, and analyte [CO_2_] concentration (z in ppm) indication
Neutral red	NR	NR(PxTyCO_2_z)
Phenol red	PR	PR(PxTyCO_2_z)
m-cresol purple	CP	CP(PxTyCO_2_z)

### Response time and pH effect

For the assay, CO_2_ (50–120 ppm concentration) was passed to the dye solutions with pH values of 4,7, and 10 at room temperature. The response time for the dyes was estimated by calculating the time from the addition of the CO_2_ to the corresponding visible color change observed in the dye solution. Neutral red, m-cresol purple, and Phenol red successfully detected CO_2_ (Table 2) only at pH 10. Moreover, each dye response time decreased with an increase in the CO_2_ concentration.

**Table 2:** Colorimetric detection of CO_2_ using different dyes

Sl. no.	Dye solution–CO_2_ mixture	Average response time (s)
1	NR(P10T25CO_2_)	15
2	PR(P10T25CO_2_)	15
3	CP(P10T25CO_2_)	10

**Fig. 1:**
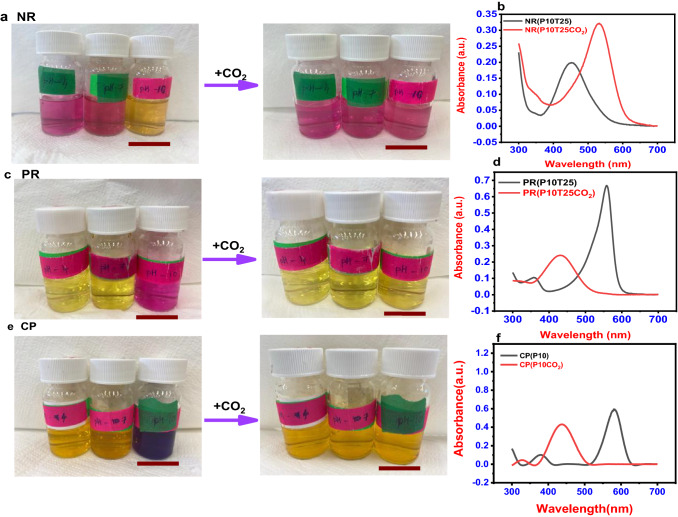
CO_2_ detection using various pH-adjusted dyes at ambient temperature. **a** Before and after adding CO_2_ to a neutral red dye solution. **b** pH 10 neutral red dye solutions UV–vis absorption spectra. **c** Before and after adding CO_2_ to phenol red dye solution. **d** pH 10 phenol red dye solutions UV–visible absorption spectra. **e** Before and after adding CO_2_ to the m-cresol purple solution. **f** pH 10 m-cresol purple dye solutions UV–vis absorption spectra

The color change in the neutral red dye solution after passing 50 ppm CO_2_ is shown in Fig. 1a, b. Figure 1a shows that with CO_2_, the color of the pH 10 neutral red dye solution tends to change from yellow to pink. For CO_2_ concentrations as low as 50 ppm, the color reaction of neutral red and CO_2_ was seen to be completed in 15 s. In the UV–vis absorbance spectra, the formation of a new absorption band centred at 533 nm with a.u.0.321 from 452 nm (a.u. 0.199) was detected (Fig. 1b).

For the CO_2_ concentration of 50 ppm, a visible color shift was observed in the pH 10 solution for the CO_2_ assay in the phenol red solution. The color shift in the phenol red solution after the addition of 50 ppm CO_2_ is shown in Fig. 1c. In a dye solution with a pH of 10, a visible color change from pink to yellow was noticed. With a rise in CO_2_ concentration, the response time of CO_2_ in the phenol red dye solution decreased, and the average response time was calculated to be 15 s. Figure 1d shows a change in the peak of the dye solution during the UV analysis from 558 nm (a.u. 0.688) to 431 nm (a.u. 0.231).

The color shift in the m-cresol purple solution after adding 50 ppm CO_2_ is shown in Fig. 1e. Within 10 s, a visible color change from purple to yellow was noticed in the dye solution at pH 10, with a peak shift (Fig. 1f) from 577 (0.536 a.u) to 435 nm (0.352a.u). At a concentration of CO_2_ as low as 50 ppm, the apparent color shift may be identified, providing a simple way to detect CO_2_ with the naked eye. The following might be the chemical reaction behind the color change in the case of m-cresol purple with CO_2_ (Elhadd et al. (2020); Rini et al. 2004; Roberts and Danckwerts 1962; Sha et al. 2022).

1 $${\text{CP}}^{{{2} - }} \left( {{\text{purple}}} \right) \, + {\text{H}}_{{2}} {\text{O}} + {\text{CO}}_{{2}} \leftrightarrow {\text{ CP}}^{{{2} - }} \left( {{\text{purple}}} \right) \, + {\text{H}}_{{2}} {\text{CO}}_{{3}} \leftrightarrow {\text{ CPH}}^{ - } \left( {{\text{yellow}}} \right) + {\text{HCO}}_{{3}}{^{ - }}$$

At varied pH levels in each dye, data were evaluated both before and after the CO_2_ was passed to the dyes. After adding CO_2_ at pH 10, these UV–Vis tests using neutral red revealed a bathochromic effect. The existence of the auxochrome (CO_2_), which may alter solvent polarity, may be the reason for the change in absorbance to a longer wavelength. The noticeable color shift provides a simple method for detecting CO_2_ with the naked eye. The UV analysis revealed hypsochromic shift in the cases of phenol red solution and m-cresol purple solution as demonstrated by a change in spectral band position in the absorption spectrum to a shorter wavelength (Geetha et al. 2022). The color of m-cresol purple showed two absorption peaks. This occurrence frequently occurs in highly conjugated systems. Nevertheless, it is frequently solvent-dependent and may result from electronic transitions between the various vibrational energy levels that each potential electronic state can support (Sha et al. 2022).

### Reusability

**Fig. 2:**
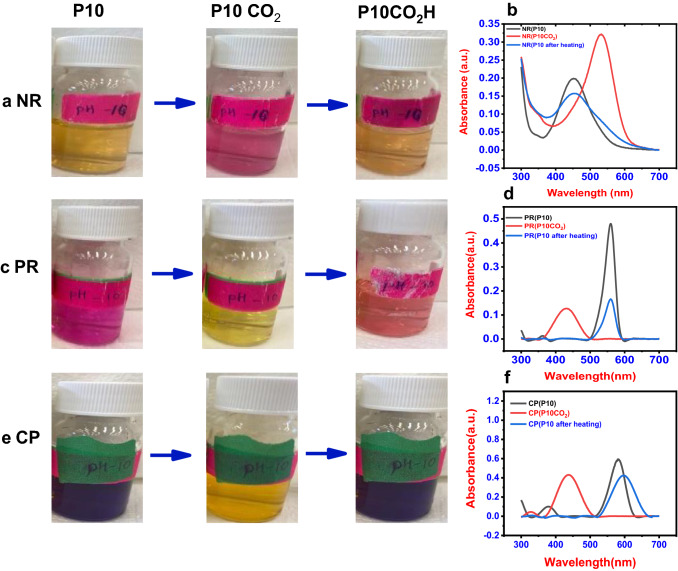
Reusability study of the dye solutions in the order of, at pH 10 (P10), with CO_2_ (P10 CO_2_), after heating (P10CO_2_H), and the corresponding UV–vis absorbance spectra respectively in each row. **a** Neutral red solution, **b** phenol red solution, **c** m-cresol purple solution

The most challenging aspects of conventional sensors based on color changes are recyclability and reusability. For cost-effectiveness and environmental safety, the sensor's reusability is crucial. Visual colorimetric sensors based on reusable substrates are well-suited for the on-site detection and monitoring of CO_2_. After passing CO_2_, the dye solutions were heated to 50 °C, and color change was observed. It is possible to eliminate the CO_2_ from the solution by boiling it. As a result, the reusability of the sensor can be tested by heating the dye solution. After 20 to 30 min of heating at 50 °C, neutral red, phenol red, and m-Cresol purple return to their native color, as seen in Fig. 2. The dye-loaded adsorbent was regenerated using microwave heating in a previous study by Arabkhani et al. (2021).

### Temperature effect

**Fig. 3:**
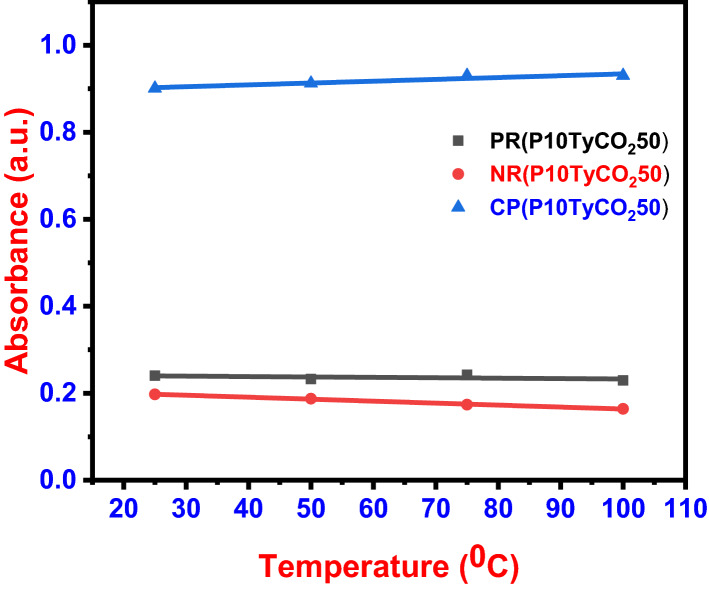
Temperature effect of dye solutions at 25, 50, 75, and 100 °C with CO_2_ (50 ppm)

When the temperature of the surrounding medium fluctuates, any biosensor should be able to keep its detecting system stable. The stability of the dye system and the physical dimension of the molecule are affected by temperature. Temperature changes raise vapo pressure while lowering response and sensitivity. As a result, the temperature should have no impact on a reliable sensor system. To study the temperature effect, the dye solutions were heated to different temperatures: 25, 50, 75, and 100 °C, and CO_2_ (50 ppm) was passed to the dye solutions. The absorption intensity remained almost constant for all dye solutions, regardless of temperature variations (Fig. 3).

Phenyl red has an absorbance variance of 0.2327 ± 0.0099, while neutral red has an absorbance variation of 0.1018 ± 0.2185, according to the findings. Cresol purple's absorbance changed similarly, near 0.9302 ± 0.0794. This implies that the multisensor system is extremely stable, which is ideal for the envisioned colorimetric sensor's real-time application. Previously, the same procedure was used to determine the temperature effect of dye-based acetone sensors, and it was discovered that temperature does not affect the dye (Sha et al. 2022).

### Sensitivity analysis

**Fig. 4:**
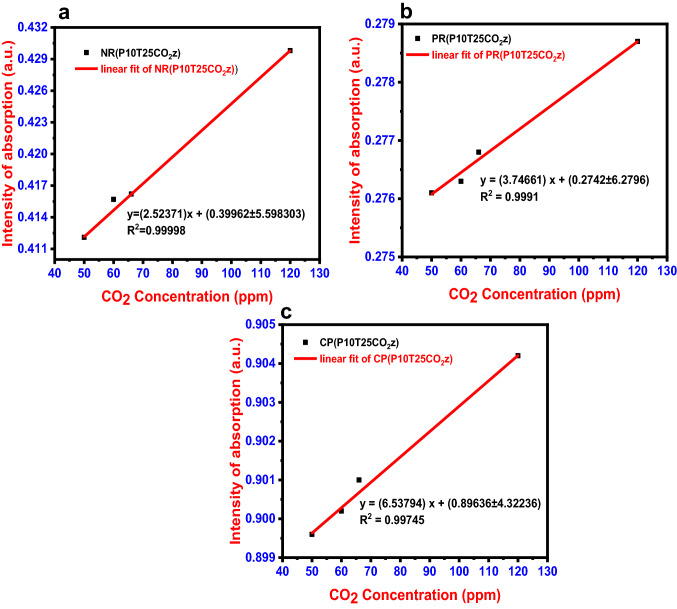
CO_2_ calibration plot for **a** Neutral Red, **b** Phenol Red, and **c** m-cresol purple

Various CO_2_ concentrations (50–120 ppm) were administered to dye solutions at room temperature to evaluate the concentration effect of dyes (Fig. 4). When the influence of concentration was investigated, it was observed that color change happened more quickly for all dye solutions when the CO_2_ was in higher concentration. Beer–Lambert law states that absorbance and concentration have a linear relationship, which can be used to predict the concentration of a solution by measuring its absorbance. The absorbance against concentration is plotted on a linear calibration curve (Fig. 4).

The LOD (Limit of Detection) was calculated using the 3*σ*/*m*, where m is the slope calibration plot and *σ* is the intercept's standard deviation which gives the lowest concentration of an analyte that can be reliably detected in a sample.

The peak absorbance of the dye was used to create the calibration curve. Figure 4 displays the CO_2_ calibration curve. Neutral red's LOD was calculated to be 7.11 ppm [*y* = (2.52371) *x* + (0.39962 ± 5.598303); *R*^2^ = 0.99998]. The calibration curve for phenol red was plotted from 50 to 120 ppm, precisely like it was for neutral red. The phenol red dye has a LOD of 5.03 ppm for CO_2_ sensing, according to the linear fit [*y* = (3.74661) *x* + (0.2742 ± 6.2796); *R*^2^ = 0.9991]. Cresol purple's calculated LOD was 1.98 ppm [*y* = (6.53794) *x* + (0.89636 ± 4.32236); *R*^2^ = 0.99745]. According to the sensitivity analysis, these three dye systems have a high sensitivity to CO_2_, with a detection limit as low as 1.98 ppm.

### Selectivity analysis

To ensure the sensor's selectivity and specificity, additional test solutions representing various biomarkers in breath, like hydrogen peroxide, ammonia, toluene, benzene, formaldehyde, and ethanol were added to the dye solutions. The concentration of the test analyte was 5 ppm, and dye solutions were tested at room temperature before being subjected to UV–vis absorption measurement. All dye solutions were very selective toward CO_2_, according to the findings. The CO_2_ selectivity of the dye was confirmed by using the equation below to calculate the relative change in wavelength (∆*λ*) from UV–vis analysis.

2 $$\left(\Delta\lambda \right)=\frac{{\lambda_{\text{x}}}-{\lambda_{0}}}{{\lambda_{0}}}\times 10,$$

where * λ*_X_ is the wavelength of absorbance peak in the presence of the analyte and * λ*_0_ is the wavelength of maximum absorbance in the blank solution. At pH 10 the value is estimated for neutral red, m-cresol purple, and phenol red.

**Fig. 5:**
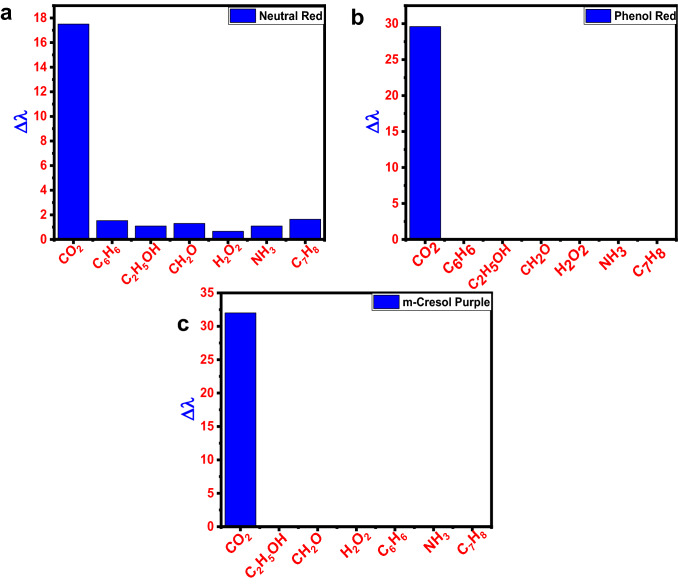
Selectivity analysis of biomarkers in different dye solutions: **a** neutral red dye solutions, **b** phenol red, and **c** m-cresol purple

Only the matching biomarkers were detected by the dye solutions (Fig. 5). These findings suggest that other interfering chemicals had little or no effect on CO_2_ colorimetric detection.

### Real-time evaluation and validation of the proposed colorimetric method

**Fig. 6:**
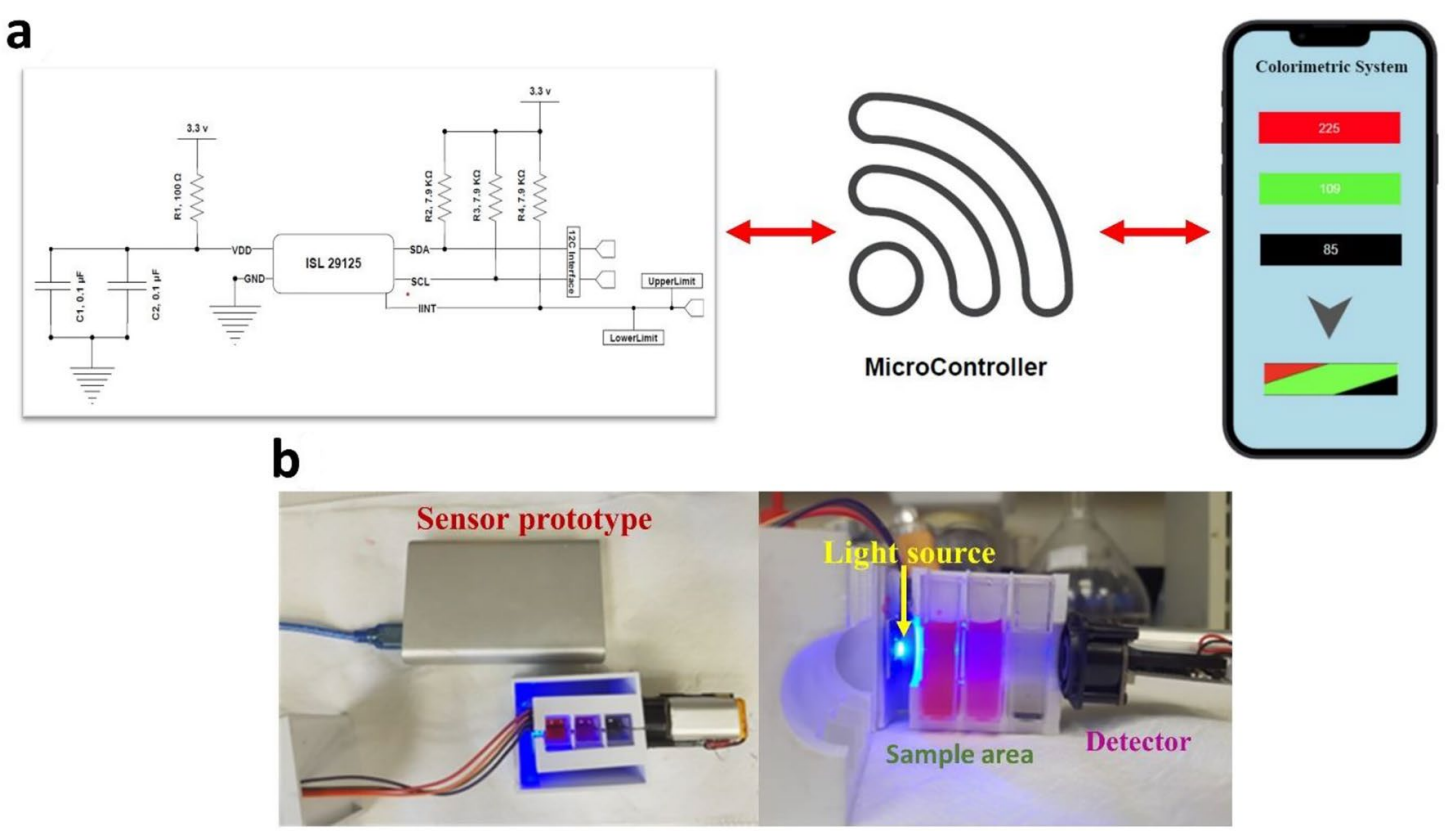
**a** Schematic of the fabricated sensor prototype, illustrating that the dye system triggered a colorimetric array and smartphone-interfaced unit to detect CO_2_ levels. **b** Real image of the developed sensor prototype

A portable prototype device with full CO_2_ detection capabilities was created (Fig. 6). When exposed to CO_2_, the sensor prototype revealed unique RGB values using dyes as sensing elements.Three dyes were chosen to detect CO_2_ at a concentration of 50 ppm in the first method, and the RGB values obtained are shown in Table 3.

**Table 3:** Analysis of CO_2_-dye mixture using sensor prototype at 50 ppm CO_2_ concentration (X1 = 50 ppm)

Sl. no.	CO_2_-dye mixture	RGB values
1	NR(Nil)PR(Nil)CP(Nil)	260, 185, 23
2	NR (CO_2_ X_1_) PR(Nil)CP(Nil)	240,185, 23
3	NR(Nil)PR (CO_2_ X_1_) CP(Nil)	260, 107, 23
4	NR(Nil)PR(Nil)CP (CO_2_ X_1_)	260, 185, 75
5	NR (CO_2_ X_1_) PR (CO_2_ X_1_) CP(Nil)	245, 110, 23
6	NR (CO_2_ X_1_) PR(Nil)CP (CO_2_ X_1_)	245, 185, 80
7	NR(Nil)PR (CO_2_ X_1_) CP (CO_2_ X_1_)	260, 110,75
8	NR (CO_2_ X_1_) PR (CO_2_ X_1_) CP (CO_2_X_1_)	238, 110, 80

CO_2_ in various concentrations using the sensor prototype in various dyes was examined in the second approach. The RBG values for various CO_2_ concentrations in various colors are presented in Table 4.

**Table 4:** Analysis of CO_2_ in different concentrations with the dyes using sensor prototype

Sl. no.	CO_2_-dye mixture at different concentrations	RGB values
1	NR(Nil)PR(Nil)CP(Nil)	260, 185, 23
2	NR(CO_2_X_2_) PR(CO_2_X_2_) CP(CO_2_X_2_)	245, 114, 30
3	NR(CO_2_X_3_) PR(CO_2_X_3_) CP(CO_2_X_3_)	251, 117, 37
4	NR(CO_2_X_4_) PR(CO_2_X_4_) CP(CO_2_X_4_)	255, 124, 45

X_1_ = 50 ppm, X_2_ = 60 ppm, X_3_ = 66 ppm, X_4_ = 120 ppm

These tests show that we can create a unique RGB chart for every dye solution in the same way. These tests demonstrated that this sensor prototype could detect the presence and concentration of CO_2_ and its concentration using RGB values (Table 5).

**Table 5:** CO_2_ concentrations and corresponding health status

CO_2_ level	Status	Symptoms	Reference
35,000–45,000 ppm	Normal	–	Geetha et al. (2022)
≥ 50,000 ppm	High	Lung diseases Cushing's syndrome Hormonal disorders Kidney disorders Alkalosis	Hawkins and Caridi (1998)
≤ 30,000	Low	Addisons disease Acidosis Ketoacidosis Shock Kidney disorders	Hawkins and Caridi (1998)

**Fig. 7:**
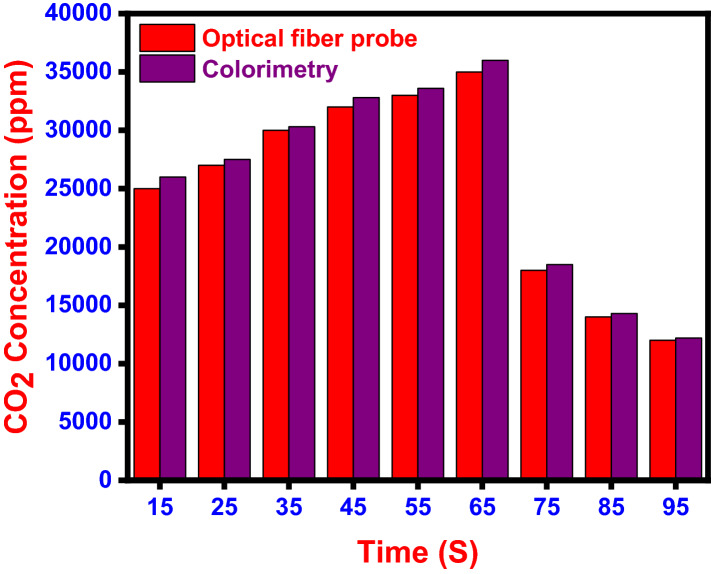
Comparison of real-time measurement of CO_2_ concentration in human breath measured at rest using optical fiber probe and colorimetry

The proposed colorimetric approach is far more cost effective than traditional detection methods in terms of cost. Our IoT-based prototype is more cost-effective instead of these expensive and heavy-duty devices (Fig. 7).

A real-time measurement of CO_2_ concentration in human breath in 1 min shows the concentration in the range of 12,000 to 35,000 ppm in optical fiber probe (Katagiri et al. 2018) and 12,200 to 36,000 ppm in this developed sensor prototype. These figures show that this novel colorimetric CO_2_ detection and quantification method would be a more precise analysis method. It can take the place of a variety of heavy-duty instruments that serve the same purpose. The sensor prototype is lightweight and may be used with any Bluetooth device (Table 6).

**Table 6:** Summary of various non-invasive sensors used for the breath CO_2_ analysis

Sl. no.	Sensors	LOD (ppm)	References
1	GC milli-whistle	3	Lin and Chen (2002)
2	Clad-etched fiber Bragg grating with polyallylamine-amino-carbon nanotube	75	Shivananju et al. (2013)
3	Ru nanobeads doped HPTS in ormosil matrix	800	Bültzingslöwen et al. (2002)
4	CNTs	75	Shivananju et al. (2013)
5	NaYF4: Yb, Er nanoparticles	1100	Ali et al. (2010)
6	Present work	1.98	Present work

## Conclusions

A non-invasive colorimetric sensor to measure CO_2_ in exhaled breath was invented for biomedical applications. In terms of pH effect, response time, reusability, concentration impact, and temperature effect, a highly selective dye system for determining CO_2_ levels was developed. Using dye solutions as sensing elements we constructed a low-cost and portable fully functional IoT-based prototype device for breath biomarker detection was created. We detected CO_2_ using the suggested multi-dye method with a detection limit of 1.98 ppm. With this apparatus, we can quickly anticipate the CO_2_ level. This novel method has a great deal of potential as a visual sensor platform in the fields of environmental and biological chemistry. This very sensitive and discriminating colorimetric sensor comes with a smartphone-assisted tool for measuring the concentration of visual biomarkers. Our sensor can also be applied to controlled environment horticulture, landfill monitoring, process control, and interior air quality monitoring, among other things.

## References

[CR1] Ali R, Saleh SM, Meier RJ, Azab HA, Abdelgawad II, Wolfbeis OS (2010). Upconverting nanoparticle based optical sensor for carbon dioxide. Sens Actuators B Chem.

[CR2] Arabkhani P, Javadian H, Asfaram A, Hosseini SN (2021). A reusable mesoporous adsorbent for efficient treatment of hazardous triphenylmethane dye wastewater: RSM-CCD optimization and rapid microwave-assisted regeneration. Sci Rep.

[CR3] Brown RH, Brooker A, Wise RA, Reynolds C, Loccioni C, Russo A, Risby TH (2013). Forced expiratory capnography and chronic obstructive pulmonary disease (COPD). J Breath Res.

[CR4] Carvajal MA, de Vargas-Sansalvador IP, Palma AJ, Fernández-Ramos MD, Capitán-Vallvey LF (2010). Hand-held optical instrument for CO_2_ in gas phase based on sensing film coating optoelectronic elements. Sens Actuators B Chem.

[CR5] Danckwerts PV, Kennedy AM, Roberts D (1963). Kinetics of CO_2_ absorption in alkaline solutions—II: Absorption in a packed column and tests of surface-renewal models. Chem Eng Sci.

[CR6] Elhadd T, Mall R, Bashir M, Palotti J, Fernandez-Luque L, Farooq F, Al Mohanadi D, Dabbous Z, Malik RA, Abou-Samra AB (2020). Artificial Intelligence (AI) based machine learning models predict glucose variability and hypoglycaemia risk in patients with type 2 diabetes on a multiple drug regimen who fast during ramadan (The PROFAST–IT Ramadan study). Diabetes Res Clin Pract.

[CR7] Elosua C, Matias IR, Bariain C, Arregui FJ (2006). Volatile organic compound optical fiber sensors: a review. Sensors.

[CR8] Fabius TM, Eijsvogel MM, Van Der Lee I, Brusse-Keizer MG, De Jongh FH (2016). Volumetric capnography in the exclusion of pulmonary embolism at the emergency department: a pilot study. J Breath Res.

[CR9] Fernandez-Sanchez JF, Cannas R, Spichiger S, Steiger R, Spichiger-Keller UE (2007). Optical CO_2_-sensing layers for clinical application based on pH-sensitive indicators incorporated into nanoscopic metal-oxide supports. Sens Actuators B Chem.

[CR10] Geetha M, Kallingal N, Sha MS, Sadasivuni KK, Sawali M, Alsaedi F, Morsy H, Ibrahim M, Ahmed AE, Abuznad R, Alruwaili A (2022). Versatile inexpensive paper-based chemosensor to detects trimethylamine: a proof of concept. Sens Actuators A.

[CR11] Guthrie BD, Adler MD, Powell EC (2007). End-tidal carbon dioxide measurements in children with acute asthma. Acad Emerg Med.

[CR12] Hawkins IF, Caridi JG (1998). Carbon dioxide (CO_2_) digital subtraction angiography: 26-year experience at the University of Florida. Eur Radiol.

[CR13] Humphreys S, Schibler A, von Ungern-Sternberg BS (2021). Carbon dioxide monitoring in children—a narrative review of physiology, value, and pitfalls in clinical practice. Pediatr Anesth.

[CR14] Jaffe MB, Orr J (2010). Continuous monitoring of respiratory flow and CO_2_. IEEE Eng Med Biol Mag.

[CR15] Kassabian J, Miller KD, Lavietes MH (1982). Respiratory center output and ventilatory timing in patients with acute airway (asthma) and alveolar (pneumonia) disease. Chest.

[CR16] Katagiri T, Shibayama K, Iida T, Matsuura Y (2018). Infrared hollow optical fiber probe for localized carbon dioxide measurement in respiratory tracts. Sensors.

[CR17] Kesten S, Maleki-Yazdi MR, Sanders BR, Wells JA, McKillop SL, Chapman KR, Rebuck AS (1990). Respiratory rate during acute asthma. Chest.

[CR18] Kostenko MA, Petrov DV, Popova MA, Tanichev AS (2021) Detection of methane in the air using a laser Raman spectrometer. In: XV international conference on pulsed lasers and laser applications, vol 12086, pp 454–458 10.1117/12.2614028.

[CR19] Kumar VS, Krishnamoorthi C (2021). Development of electrical transduction based wearable tactile sensors for human vital signs monitor: fundamentals, methodologies and applications. Sens Actuators A.

[CR20] Lin YS, Chen YC (2002). Laser desorption/ionization time-of-flight mass spectrometry on sol–gel-derived 2, 5-dihydroxybenzoic acid film. Anal Chem.

[CR21] Lin CH, Wu LX, Chen KH, Lo HF, Lin KC, Kasai T, Chen CC, Shih CH, Manzano MC, Santos GN, Manzano E (2020). Non-invasive and time-dependent blood sugar monitoring via breath-derived CO_2_ correlation using gas chromatograph with milli-whistle gas analyzer. Anal Sci.

[CR22] Maestri R, Bruschi C, Olmetti F, La Rovere MT, Pinna GD (2013). Assessment of the peripheral ventilatory response to CO_2_ in heart failure patients: reliability of the single-breath test. Physiol Meas.

[CR23] Mannino DM, Homa DM, Akinbami LJ, Ford ES, Redd SC (2002) Chronic obstructive pulmonary disease surveillance-United States. 1971–2000. 10.1016/j.jinf.2021.05.019 12198919

[CR24] Nagurka R, Bechmann S, Gluckman W, Scott SR, Compton S, Lamba S (2014). Utility of initial prehospital end-tidal carbon dioxide measurements to predict poor outcomes in adult asthmatic patients. Prehosp Emerg Care.

[CR25] Nakamura N, Amao Y (2003). Optical sensor for carbon dioxide combining colorimetric change of a pH indicator and a reference luminescent dye. Anal Bioanal Chem.

[CR26] Ottonello-Briano F, Errando-Herranz C, Rödjegård H, Martin H, Sohlström H, Gylfason KB (2020). Carbon dioxide absorption spectroscopy with a mid-infrared silicon photonic waveguide. Opt Lett.

[CR27] Qamar S, Torres YJ, Parekh HS, Falconer JR (2021). Extraction of medicinal cannabinoids through supercritical carbon dioxide technologies: a review. J Chromatogr B.

[CR28] Rasera CC, Gewehr PM, Domingues AM (2015). PETCO_2_ measurement and feature extraction of capnogram signals for extubation outcomes from mechanical ventilation. Physiol Meas.

[CR29] Rini M, Pines D, Magnes BZ, Pines E, Nibbering ET (2004). Bimodal proton transfer in acid-base reactions in water. J Chem Phys.

[CR30] Roberts D, Danckwerts PV (1962). Kinetics of CO_2_ absorption in alkaline solutions—I. Transient absorption rates and catalysis by arsenite. Chem Eng Sci.

[CR31] Sha MS, Maurya MR, Shafath S, Cabibihan JJ, Al-Ali A, Malik RA, Sadasivuni KK (2022). Breath analysis for the in vivo detection of diabetic ketoacidosis. ACS Omega.

[CR32] Shivananju BN, Yamdagni S, Fazuldeen R, Sarin Kumar AK, Hegde GM, Varma MM, Asokan S (2013). CO_2_ sensing at room temperature using carbon nanotubes coated core fiber Bragg grating. Rev Sci Instrum.

[CR33] Tidemandsen C, Hansen ES, Rasmussen SM, Ulrik CS, Backer V (2021). Unique aspects of asthma in women. Clin Chest Med.

[CR34] von Bültzingslöwen C, McEvoy AK, McDonagh C, MacCraith BD, Klimant I, Krause C, Wolfbeis OS (2002). Sol–gel based optical carbon dioxide sensor employing dual luminophore referencing for application in food packaging technology. Analyst.

[CR35] Williams E, Dassios T, O’Reilly N, Walsh A, Greenough A (2021). End-tidal capnography monitoring in infants ventilated on the neonatal intensive care unit. J Perinatol.

[CR36] Yaron M, Padyk P, Hutsinpiller M, Cairns CB (1996). Utility of the expiratory capnogram in the assessment of bronchospasm. Ann Emerg Med.

[CR37] Zhao D, Miller D, Xian X, Tsow F, Forzani ES (2014). A novel real-time carbon dioxide analyzer for health and environmental applications. Sens Actuators B Chem.

